# Brain-derived neurotrophic factor (Val66Met) polymorphism and olfactory ability in young adults

**DOI:** 10.1186/1423-0127-20-57

**Published:** 2013-08-07

**Authors:** Alessandro Tonacci, Andrea Borghini, Antonella Mercuri, Giovanni Pioggia, Maria Grazia Andreassi

**Affiliations:** 1 Institute of Clinical Physiology, National Research Council (IFC- CNR), Pisa, Italy

**Keywords:** Olfactory function, Brain - derived neurotrophic factor, Val66Met polymorphism

## Abstract

**Background:**

Brain- derived neurotrophic factor (BDNF) is linked to neurodegenerative diseases (e.g. Alzheimer disease and Parkinson disease) which are often characterized by olfactory impairment. A specific single nucleotide polymorphism of the BDNF gene, the Val66Met, modulates intracellular trafficking and activity-dependent secretion of BDNF protein. The aim of this study was to investigate a possible association between brain- derived neurotrophic factor Val66Met polymorphism and olfactory function, a well-known biomarker for neurodegeneration, in healthy young adults. A total of 101 subjects (45 males, age 38.7 ± 9.4 years) were assessed using the Sniffin’ Sticks Extended Test, a highly reliable commercial olfactory test composed of three sub-parts, calculating olfactory threshold (sensitivity), odor discrimination and odor identification. The Val66Met polymorphism was determined by polymerase chain reaction -restriction fragment length polymorphism (PCR-RFLP) analysis.

**Results:**

An impaired function in Met carriers was found, especially when compared to subjects with Val/Val genotype, in the threshold (5.5 ± 2.0 vs 6.5 ± 1.8, p = 0.009), discrimination (10.3± 2.5 vs 11.9 ± 2.2, p = 0.002), and identification task (13.3 ± 1.6 vs 14.1 ± 1.3, p = 0.007), as well as in the overall TDI Score (29.1 ± 4.5 vs 32.6 ± 3.9, p < 0.001).

**Conclusions:**

These findings appear to have implications for the evaluation of olfactory function and the relation of its impairment to cognitive decline and neurodegenerative disease.

## Background

Olfactory function is a well-known biomarker for neurodegeneration. The loss of olfaction is, in fact, often associated with many neurodegenerative conditions, such as Parkinson’s (PD) and Alzheimer’s (AD) diseases [1,2]. A decrease in olfactory function could be an early alarm warning for such conditions, since its onset occurs prior to the first clinical signs of pathology by 4–5 years. Many factors are known to influence olfactory ability, including genetic variability [3,4]. Moreover, an increasing number of animal studies, as well as clinical studies, confirm the important role of the brain- derived neurotrophic factor (BDNF) in neurodegenerative diseases [5-9]. In particular, BDNF, a member of the neurotrophin family, plays an important role in the development and maintenance of neurons and neuronal connections in the central and peripheral nervous system [10]. A common functional single nucleotide polymorphism of the BDNF gene, the Val66Met, modulates intracellular trafficking and activity-dependent secretion of BDNF protein, and impairs the ability of BDNF to undergo activity-dependent release, but not general secretion [11-14]. The Val66Met is located at nucleotide 196 (G/A) in the pro-protein of BDNF and produces an amino acid substitution, valine to methionine. The Met allele inhibits the intracellular trafficking and the regulated secretion of the BDNF protein at synapses [11]. Published data support a role for BDNF gene variant in some neuropsychiatric disorders. Guerini et al. showed a correlation between BDNF Val66Met polymorphism and cognitive impairment in Italian patients with Parkinson’s disease [15]. BDNF Val66Met is also reported to be associated with AD and/or schizophrenia, as well as other psychoses [16,17], but the findings are still contradictory [18,19]. Furthermore, evidence suggested that BDNF might be relevant for olfactory processing [20]. In fact, BDNF induces the proliferation and survival of neuronal precursor cells/immature neurons in the olfactory system *in vivo* and *in vitro* using mice with targeted deletion of the gene for BDNF and an olfactory epithelium culture system, respectively [20]. A recent work found an association between mature BDNF levels and increasing of mitral cell, pyramidal-like neurons, excitability olfactory bulb level in the mice [21]. Furthermore, an experimental study showed that BDNF play a pivotal role in the olfactory neurogenesis, contributing in olfactory epithelium to the early stage of regeneration and in olfactory bulb in the late stage of regeneration of olfactory receptor neurons [22]. Additionally, there are experimental studies in animals linking the genetic variant to olfactory function [23], but, to date, little is known about this association in humans, except for a recent population-based study that showed a link between age-related olfactory decline and BDNF Val66Met polymorphism in the older age cohort (70–90 years) [24]. In order to better define the influence of BDNF Val66Met polymorphism on the olfactory function, the purpose of this study was to assess a possible association between BDNF Val66Met polymorphism and olfactory ability in healthy young adults, population less subjected to comorbidities possibly associated with an olfactory dysfunction if compared with elderly people.

## Methods

### Ethics

The present study was approved by the Ethics Committee for clinical trials with medicines - Pisa Hospital, Italy, with protocol number 36169.

### Study population

A total of 101 volunteers (45 males, age 38.7 ± 9.4 years) were enrolled for the study. Written informed consent was obtained from all subjects. The volunteers were chosen from among young adults, as this population is less subject to diseases affecting olfactory function. A clinical questionnaire was filled out by the volunteers and collected. Many exclusion criteria were applied to obtain a clean population, with absence of pathological conditions possibly affecting the sense of smell. In particular, subjects having nasal problems, such as flu, rhinitis, sinusitis, allergies, were excluded from the analysis, together with subjects having used medications such as nasal decongestants, antidepressants and anxiolytics in the 2-month period before administration of the olfactory test. Familiar history of neurodegeneration was also considered, and subjects with parents or first degree relatives with conditions such as Alzheimer’s disease, Parkinson’s disease, Amyotrophic Lateral Sclerosis, Lewy body disease, Huntington’s chorea or schizophrenia were excluded from the study. Among normal subjects matching these criteria, people with normal general cognitive function evaluated with standardized neuropsychological test batteries by a professional neuropsychologist were included, while subjects with sub-normal scores were not considered in the analysis.

### Olfactory assessment

Olfactory function was assessed using the Sniffin’ Sticks Extended Test [25,26], an olfactory test commercially distributed by Burghart, Medizintechnik, GmbH (*Wedel*, *Germany*). It consists of three different sub-tests, assessing the olfactory sensitivity (threshold), discrimination and identification, typical tasks of the olfactory system. In this version of the test, the olfactory sensitivity to n-butanol was employed. The olfactory threshold is considered as the minimum concentration of an odorant (n-butanol) that can be detected by a subject. N-butanol was presented in 16 different dilutions in felt tip pens. For each trial the blindfolded subject was subjected to three different stimuli, one consisting of a given concentration of n-butanol, and the other two with blank stimuli. The subject was asked which of the three stimuli contained the n-butanol (or, equivalently, which of the three stimuli was the strongest). Depending upon the correct and wrong answers given, the concentration of the stimulus was changed and the trial was repeated up to seven staircase reversals. The threshold score was calculated by performing a mean of the values of four last reversals. The olfactory discrimination’s aim was to assess the subject’s ability to discriminate between different odorants. Even in this case the subject was blindfolded and 16 different triplets of odorants were presented. For each triplet, two felt tip pens contained the same odorant, while the third one held a different substance. The subject was asked which of the three pens contained the different odorant. The olfactory identification test aimed to evaluate the subject’s ability to correctly identify an odorant. The subject was presented with16 different odorants and asked to identify them by choosing between four possible odors for each trial. In this final test, the subject was not blindfolded. Each of these tests yielded a score, and the total sum of the three sub-scores was called “TDI (Threshold Discrimination Identification) Score”, relating to olfactory function. We chose to employ a bilateral testing, in order to avoid possible false results due to the congestion of one of the two nostrils, even though the presence of flu and/or nasal problems was included in the exclusion criteria of the survey. The test was performed once for each participant, given the high test-retest reliability of the method employed (r = 0.80 for Odor Discrimination, r = 0.88 for Odor Identification, r = 0.92 for Odor Threshold) [25,27]. The reliability data obtained in previous pilot studies are in agreement with data above mentioned.

### DNA extraction and genotyping

Genomic DNA was extracted from peripheral blood leukocytes. The BDNF Val66Met polymorphism was genotyped by polymerase chain reaction amplification and restriction enzyme digestion, as previously described [28]. Briefly, a 274 bp DNA segment including the polymorphic site was amplified by PCR using a set of oligonucleotide primers: 5’-AAA GAA GCA AAC ATC CGA GGACAA G-3’ and 5’-ATT CCT CCA GCA GAA AGA GAA GAG-3’, sense and antisense primers respectively. The PCR product was digested with NlaIII restriction endonuclease, resulting in two fragments of 57 and 217 bp for the G allele and in three fragments of 57, 77, 140 bp for the A allele. The products were separated by 2% agarose gel stained with ethidium bromide. Genotype results were regularly confirmed by random repetition of the samples with no discrepancies.

### Statistical methods

Statistical analysis of the data was performed with the StatView statistical package, version 5.0.1 (*Abacus Concepts*, *Berkeley*, *CA*, *USA*). Data are expressed as mean ± SD. Student’s T-test was used to examine demographic data and to compare individual’s genotypes with olfactory tests called “Threshold Test”, “Discrimination Test” and “Identification Test”, as well as with the overall “TDI Score”. Fisher’s exact T test was used for post-hoc tests. The level of significance was set at p < 0.05 for all statistical analyses. Assuming a frequency of 23% for the risk allele, the size of the study population allows to detect a 15% difference or more in olfactory parameters in the heterozygous carriers of Met variant the with a power of β = 80% by means of a two-sided *t*-test with α = 5%.

## Results

**Figure 1:**
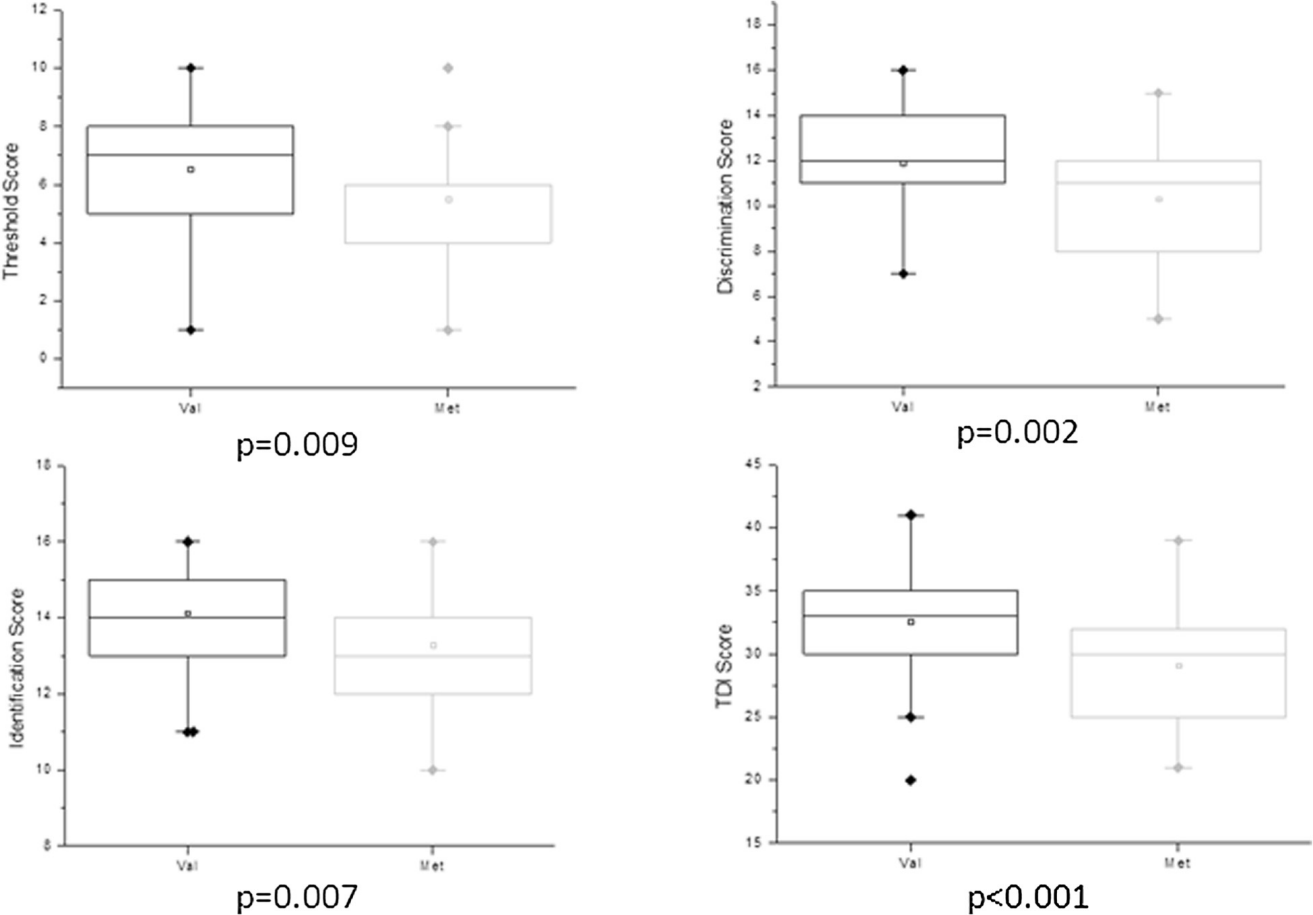
**Impact of the BDNF Val66Met polymorphism on olfactory ability.** BDNF Val66Met polymorphism and the olfactory threshold (*top left*), discrimination (*top right*), identification (*bottom left*) and the overall TDI Score (*bottom right*).

We investigated a possible association of BDNF Val66Met polymorphism with olfactory function in a group of young adults. Due to the low population frequency of the Met/Met genotype (<5%), participants were divided into two groups, either homozygous for the Val allele (Val/Val) or homozygous and heterozygous for the Met allele (Met/Met, Val/Met), respectively. The demographic characteristics of the study population are reported in Table 1. There was no significant difference in age, gender and smoking habits between groups. The genotype’s distribution of Val66Met polymorphism observed in both patients and controls satisfied the Hardy-Weinberg equilibrium and were comparable with that previously observed in Caucasian subjects [11]. There was significant evidence for the impact of the BDNF Val66Met polymorphism on olfactory ability. In particular, Met carriers showed impaired olfactory function compared with Val/Val carriers in all three sub-tests, as well as in the overall TDI Score. The impairment was marked in all tasks, in the case of Olfactory Threshold (5.5 ± 2.0 vs 6.5 ± 1.8, p = 0.009), Olfactory Discrimination (10.3 ± 2.5 vs 11.9 ± 2.2, p = 0.002), and Identification (13.3 ± 1.6 vs 14.1 ± 1.3, p = 0.007), as well as in the TDI Score (29.1 ± 4.5 vs 32.6 ± 3.9, p < 0.001), suggesting a clear effect of the Val/Met variant on olfactory function, as displayed in Figure 1*.*

**Table 1:** Demographic characteristics of study population

	**Val/Val n** **=** **60**	**Met carriers n** **=** **41**
**Age (mean** **±** **SD) (years)**	38.1 ± 8.5	39.7 ± 10.7
**Gender, males n (%)**	24 (40)	21 (51)
**Smoking habit, n (%)**	17 (28)	12 (29)

## Discussion

The study aimed to discover the influence of brain- derived neurotrophic factor (BDNF) single nucleotide polymorphism (SNP) Val66Met on olfactory function. Brain- derived neurotrophic factor (BDNF) is widely expressed in the adult hippocampus and neocortex [29]. It is considered an important neurotrophic factor for neuronal differentiation and life-long plasticity and repair [30]. BDNF Val66Met polymorphism impairs activity-dependent BDNF excretion and has been associated with changes in cortical and subcortical anatomy [31,32]. BDNF Val66Met polymorphism is strictly related to cognitive function. In particular, Met carriers show impaired ability in declarative memory tasks, as well as a decrease in engagement of particular areas, such as the hippocampus, during encoding and retrieval [33], although current findings are still contradictory [34,35], and a recent meta-analysis reported no significant correlation between Val66Met SNP and the cognitive phenotypes [36]. BDNF also appears to influence the olfactory function, since the modulation of proliferation and survival of olfactory receptors is one of its key-roles, according to Simpson *et al*. [20]. The expression of BDNF Val66Met SNP is relevant in particular areas, involved in olfactory processing and part of the olfactory pathway, such as in the olfactory bulb, whose neurogenesis disruption is strictly related to the BDNF Val66Met variant. To date, only one work has been published showing an association between olfactory function (identification task) and BDNF Val66Met SNP in humans [24], but the effect of this SNP in young adults was not investigated, nor was its effect on other important tasks, such as olfactory discrimination. Furthermore, we chose to employ the complete Sniffin’ Sticks Extended Test in order to evaluate eventual variations occurring at different levels of the olfactory pathway. In particular, a clear impairment for Met carriers was found in all tasks, suggesting the profound influence of this genetic variant on the good functioning of the olfactory pathway. Several key limitations need to be acknowledged. First, although the present study seems to be sufficiently powered, our sample size is relatively small, thus making statistical estimations less robust. Second, the frequencies of the Val and Met alleles of BDNF Val66Met vary by ethnicity; about 80% of the European population, but only 50% of the Asian population, carry it [16]. Another limitation of our study is the lack of a more comprehensive genetic analysis of polymorphisms potentially associated with olfactory function [37].

## Conclusions

Despite these limitations, we were able to show a relevant influence of BDNF Val66Met polymorphism on olfactory function. These findings could have implications for the evaluation of olfactory function and for relating its impairment to cognitive decline and neurodegenerative diseases. Further investigation are needed to substantiate this relationship in larger subgroups of populations of different ethnic backgrounds.

## Competing interests

The authors declare no potential conflict of interests.

## Authors’ contributions

AT, AB and AM carried out the major experiments. AT, AB and MGA contributed to the experimental design and wrote the manuscript. All authors read and approved the final manuscript.
